# Chylous ascites after nephrectomy without lymphadenectomy for malignant rhabdoid tumor of the kidney: A rare occurrence and literature review

**DOI:** 10.4103/0971-9261.59605

**Published:** 2009

**Authors:** Takahiro Einama, Tadao Okada, Fumiaki Sasaki, Satoru Todo

**Affiliations:** 1^st^ Surgery, Hokkaido University Graduate School of Medicine, Sapporo, Japan; 1 Pediatric Surgery, Hokkaido University Graduate School of Medicine, Sapporo, Japan

**Keywords:** Chylous ascites, malignant rhabdoid tumor of the kidney, nephrectomy

## Abstract

Chylous ascites (CA) is an extremely rare complication of abdominal surgery in children. This report describes a 4-month-old girl with malignant rhabdoid tumor of the kidney (MRTK), who developed CA after left nephrectomy without lymphadenectomy, and who was successfully treated conservatively with enteral therapy. The literature on CA after nephrectomy without lymphadenectomy for MRTK is reviewed herein, and the clinical problems of postoperative CA are discussed.

## INTRODUCTION

Chylous ascites (CA) is a rare condition mostly caused by the diseases that interfere with the abdominal or retroperitoneal lymphatic glands. Postoperative CA always represents a difficult problem in patient treatment. Patients are further debilitated by the serious mechanical, nutritional, and immunological consequences of the constant loss of protein and lymphocytes.[1]

We report a first case of CA after left nephrectomy without lymphadenectomy for stage IV malignant rhabdoid tumor of the kidney (MRTK) to our knowledge. The pathophysiology of occurrence, possible etiologic factors, and principles of management of CA after nephrectomy without lymphadenectomy for MRTK are discussed.

## CASE REPORT

**Figure 1:**
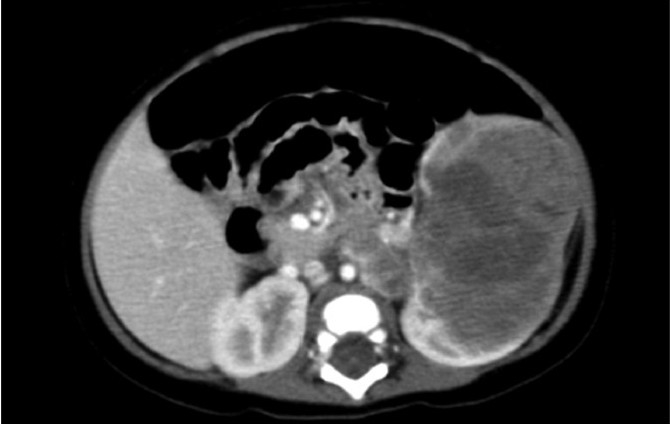
Preoperative abdominal enhanced computed tomography (a large solid mass can be noted in the left kidney; the abdominal lymph nodes were not swollen)

A 4-month-old girl was referred to our hospital for further evaluation of a large left abdominal mass and a right lung mass. Physical examination revealed a well-nourished infant, and a large solid mass was felt in the left upper quadrant of the abdomen. The mass did not cross over the midline, and its surface was smooth. Enhanced computed tomography showed neither swelling of abdominal lymph nodes nor metastases in the liver [Figure 1]. Chest radiography and computed tomography revealed metastasis to bronchopulmonary segment 5 in the right lung. These radiographic images and clinical findings suggested Wilms' tumor with lung metastasis.

The patient underwent left nephrectomy by laparotomy. The left renal artery and vein were resected with the renal hilum. Because there was no hilar and periaortic lymph node, lymph node sampling was not performed. An abdominal drainage tube was inserted into the renal bed. Histopathologic examination revealed a diagnosis of MRTK.

On postoperative day 3, we initiated oral intake, and slight abdominal distension developed. A total of 40 ml of milky fluid was aspirated from the left abdominal cavity, and was found to exhibit the chemical characteristics of chyle (triglyceride level of 632 mg/dl, and total protein 2.1 g/dl).

We then discontinued oral intake and started peripheral parenteral nutrition. On the seventh postoperative day, the chylous drainage decreased to 10 ml per day, and the triglyceride level in chyle was reduced. Limited oral feeding, consisting mainly of medium chain triglyceride (MCT) formula, was then started. She was maintained on MCT formula for 3 days. By the 10th postoperative day, the level of triglycerides in ascites returned to within the normal range (triglyceride level of 100 mg/dl, and total protein 3.3 g/dl). The patient has been maintained on an MCT formula for 1 month, and has experienced no recurrence of CA.

## DISCUSSION

The development of postoperative CA has been described infrequently in the pediatric field. As in adults, the usual cause is operative injury to the thoracic duct, cisterna chyli, or their major tributaries, and another contributing factor may be obstruction of the abdominal lymphatic channels due to malignancy.[2]

Anatomically, CA results from a rent in the thoracic duct, cisterna chyli, or its intestinal tributaries. The cisterna chyli is located at the level of the renal hilum and may be disrupted during the resection of retroperitoneal lymph nodes.[3] Lymph node dissection is not needed during nephrectomy for Wilms' tumor and MRTK, but lymph node sampling or biopsy is recommended.[4] In the present case, because there was no hilar and periaortic lymph node, lymph node sampling was not performed and we undertook a simple left nephrectomy without lymphadenectomy. We identified only two previous patients with CA after nephrectomy of MRTK without lymphadenectomy.[5,6] These and the present patient have few characteristics in common [Table 1].

**Table 1:** Postoperative chylous ascites after nephrectomy without lymphadenectom

Reference	Diagnosis	Age at operation/sex	Nephrectomy side	Interval between RS and CA diagnosis	Radiation therapy	Management
Weiser *et al*.[5]	Wilms' tumor	2 Years/M	Rt	12 days	Yes	TPN, therapeutic paracentesis, peritoneovenous shunt
Mladinic-Vulic *et al*.[6]	Hydronephrosis	6 Days/M	Rt	46 days	No	MCT
Einama *et al*. (present case)	Malignant rhabdoid tumor	4 Months/F	Lt	3 days	Yes	MCT

RS : Radical surgery

The reason for these cases of postoperative CA after nephrectomy without lymphadenectomy remains unknown. In the present case, lymphatic leakage was likely to have resulted from disruption of the lymphatic tracts, for example, during mass ligation, or due to overusing an electric surgical knife. Additionally, the cisterna chyli, which is located at the level of the renal hilum, may be disrupted during resection of the renal artery and vein in the renal hilum.

If the postoperative clinical course leads to the suspicion of chylous leakage or the accumulation of CA, diagnostic and therapeutic paracentesis is usually indicated. After the diagnosis of CA is established, most patients are placed on nonsurgical therapy. Aalami *et al*. performed a retrospective collective review of case reports of 156 patients with CA due to various causes.[7] Of these cases, 33% were managed successfully by surgery, while 67% were resolved after conservative treatment. The mainstay of nonoperative therapy for CA is an MCT diet and total parenteral nutrition to achieve a decreased lymph flow in the major lymphatic tracts and facilitate the closure of chylous fistulas. Surgical treatment, including direct repair of leaking lymphatic glands and peritoneovenous shunt placement, should be reserved for the patients in whom conservative management has been used for at least 4 weeks.[8,9]

In summary, we need to consider that CA can occur after nephrectomy in an infant even without lymph adenectomy as a complication.
